# Supplementary material for: Modeling glioblastoma heterogeneity as a dynamic network of cell states
Source: Mol Syst Biol. 2021 Sep 16;17(9):e10105. doi: 10.15252/msb.202010105 (PMC8444284; doi:10.15252/msb.202010105)
Supplement: Supplementary file 5 — Source Data for Figure 3 [file MSB-17-e10105-s001.zip › Figure3A_sourcedata/GSEA_3065/nosigdb_state6.GseaPreranked.1622718443993/DOROSZKO_U3013MG_CLUSTER_0.html]

Details for gene set DOROSZKO\_U3013MG\_CLUSTER\_0[GSEA]

|  || Dataset | state6 |
| Phenotype | NoPhenotypeAvailable |
| Upregulated in class | na\_neg |
| GeneSet | DOROSZKO\_U3013MG\_CLUSTER\_0 |
| Enrichment Score (ES) | -0.7196731 |
| Normalized Enrichment Score (NES) | -2.2290263 |
| Nominal p-value | 0.0 |
| FDR q-value | 0.0 |
| FWER p-Value | 0.0 |
Table: GSEA Results Summary

  

Fig 1: Enrichment plot: DOROSZKO\_U3013MG\_CLUSTER\_0      
 Profile of the Running ES Score & Positions of GeneSet Members on the Rank Ordered List

  

| PROBE | GENE SYMBOL | GENE\_TITLE | RANK IN GENE LIST | RANK METRIC SCORE | RUNNING ES | CORE ENRICHMENT || 1 | SNX22 |  |  | 627 | 0.112 | -0.0418 | No |
| 2 | MAP2 |  |  | 917 | 0.090 | -0.0508 | No |
| 3 | CAMK2N1 |  |  | 1346 | 0.069 | -0.0811 | No |
| 4 | CKB |  |  | 1359 | 0.069 | -0.0642 | No |
| 5 | MEF2C |  |  | 1785 | 0.054 | -0.0984 | No |
| 6 | CAVIN1 |  |  | 1909 | 0.050 | -0.0990 | No |
| 7 | NGFR |  |  | 2867 | 0.025 | -0.2015 | No |
| 8 | MAP1B |  |  | 2893 | 0.024 | -0.1980 | No |
| 9 | SYT11 |  |  | 4256 | -0.004 | -0.3521 | No |
| 10 | PCSK1N |  |  | 4606 | -0.011 | -0.3891 | No |
| 11 | SNX1 |  |  | 6853 | -0.060 | -0.6291 | No |
| 12 | IGFBP5 |  |  | 7011 | -0.065 | -0.6297 | No |
| 13 | NOVA1 |  |  | 7506 | -0.082 | -0.6644 | No |
| 14 | DMD |  |  | 7992 | -0.107 | -0.6914 | Yes |
| 15 | BCHE |  |  | 8174 | -0.119 | -0.6803 | Yes |
| 16 | PGM2L1 |  |  | 8304 | -0.132 | -0.6599 | Yes |
| 17 | GSN |  |  | 8339 | -0.137 | -0.6273 | Yes |
| 18 | ANXA1 |  |  | 8651 | -0.213 | -0.6061 | Yes |
| 19 | VCAN |  |  | 8664 | -0.219 | -0.5494 | Yes |
| 20 | NES |  |  | 8677 | -0.227 | -0.4905 | Yes |
| 21 | COL6A1 |  |  | 8698 | -0.238 | -0.4296 | Yes |
| 22 | IGFBP2 |  |  | 8719 | -0.260 | -0.3629 | Yes |
| 23 | ANXA2 |  |  | 8741 | -0.279 | -0.2912 | Yes |
| 24 | PTPRZ1 |  |  | 8783 | -0.360 | -0.2004 | Yes |
| 25 | MEG3 |  |  | 8786 | -0.370 | -0.1022 | Yes |
| 26 | TNC |  |  | 8787 | -0.389 | 0.0010 | Yes |
Table: GSEA details [plain text format]

  

Fig 2: DOROSZKO\_U3013MG\_CLUSTER\_0: Random ES distribution      
 Gene set null distribution of ES for **DOROSZKO\_U3013MG\_CLUSTER\_0**
